# Transarterial chemoembolization (TACE) with degradable starch microspheres (DSM) in hepatocellular carcinoma (HCC): multi-center results on safety and efficacy

**DOI:** 10.18632/oncotarget.19997

**Published:** 2017-08-07

**Authors:** Andreas Schicho, Philippe L. Pereira, Michael Haimerl, Christoph Niessen, Katharina Michalik, Lukas P. Beyer, Christian Stroszczynski, Philipp Wiggermann

**Affiliations:** ^1^ Department of Radiology, University Hospital Regensburg, Regensburg, Germany; ^2^ Department of Radiology, Minimal-Invasive Therapies and Nuclear Medicine, SLK Kliniken Heilbronn, Heilbronn, Germany

**Keywords:** TACE, HCC, embolization, degradable starch microspheres, safety

## Abstract

**Background:**

Hepatocellular carcinoma (HCC) is the 3rd leading cause of cancer-related death worldwide. The majority of HCCs are diagnosed in a stage that is not eligible for curative resection. For intermediate stage HCC, transarterial chemoembolization (TACE) is the recommended treatment. We evaluated the safety and efficacy of DSM (degradable starch microspheres) as embolic agent in transarterial chemoembolization (TACE) for the treatment of intermediate stage, non-resectable hepatocellular carcinoma (HCC).

**Methods and Findings:**

A national, multi-center observational study on the safety and efficacy of DSM-TACE for the treatment of intermediate HCC was conducted. The recruitment period for the study was from January 2010 to June 2014. The primary endpoints were safety and treatment response according to the mRECIST criteria.

A total of 179 DSM-TACE procedures in 50 patients were included in the analysis. The therapeutic efficacy assessed with mRECIST was as follows: complete response (n=1; 2 %), 21 partial response (42 %), 13 stable disease (26 %), 9 progressive disease (18 %), and 6 incomplete data (12 %). Thus, the objective response rate was 44% (n=22) and disease control rate was 70% (n=35).

A total of 76 immediate adverse events (AE) and 2 severe adverse events (SAE) were recorded. Forty-eight percent of patients (n=24) did not encounter any immediate AE/SAE. Between treatments, a total of 66 AE and one SAE were recorded. Twenty-four patients (48 %) did not encounter any AE/SAE in between treatments.

**Conclusion:**

The use of DSM as a TACE embolic agent appears to be safe for the treatment of HCC and has promising efficacy.

## INTRODUCTION

Hepatocellular carcinoma (HCC) is the 5^th^ most common cancer and 3^rd^ leading cause of cancer-related death worldwide [1]. The majority of HCCs are diagnosed in a stage that is not eligible for curative resection, i.e., intermediate or advanced stage HCC. For intermediate stage HCC, transarterial chemoembolization (TACE) is the recommended treatment [2]. A survival benefit has been demonstrated for TACE-treated HCC over the best supportive care [3] alone.

Different embolic agents are available for use in the TACE procedure. TACE using Lipiodol is the most common technique for the treatment of HCC [4]. The use of drug eluting beads (DEB) offers another safe and efficient treatment option, even in advanced HCC [5–7]. Conventional Lipiodol-TACE has two major drawbacks: (1) it has ill-defined and prolonged vascular occlusion over a half-time from 5 to 12 weeks [4] and (2) has lipophilic characteristics, while the most used chemotherapeutic agents, e.g., doxorubicin, are hydrophilic. The embolic agent DEB shows a well-calculable binding and release of chemotherapeutics, but causes a permanent vascular occlusion [7]. The duration of the occlusion is of importance since the dependent release of vascular endothelial growth factor (VEGF) can promote metastatic seeding. Degradable starch microspheres (DSM), such as EmboCept (PharmaCept GmbH, Berlin, Germany), combine a well-defined transient vascular occlusion and optimized binding capacities for chemotherapeutic agents [8, 9]. This study presents results on the clinical efficacy, toxicity, and safety of DSM-TACE using EmboCept microspheres for the treatment of intermediate stage HCC.

## RESULTS

Fifty patients were included between January 2010 and March 2015. They received a total of 179 DSM-TACE procedures. The youngest patient was 44 years old upon inclusion in the study, and the oldest patient was 83 years old (mean ± SD = 67.7 ± 10 years; Table 1). HCC was surgically pre-treated in 4 patients (8.0 %) before chemoembolization, and one patient (2.0 %) underwent radiofrequency-ablation (RFA). HCC metastases were not surgically pre-treated in any patients. Systemic chemotherapy preceded DSM-TACE in 2 patients (4.0 %).

**Table 1:** Baseline characteristics of patients, pre-treatment, and disease extent

Age (mean ± SD; min; max)	67.7 ± 10; 44; 83 years
Pre-treatment	
Surgery	4/50 (8.0 %)
RFA	1/50 (2.0 %)
Chemotherapy	2/50 (4.0 %)
Disease extent	
< 4 hepatic metastases	28/50 (56.0 %)
≥ 4 hepatic metastases	17/50 (34.0 %)
no. of metastases unknown	5/50 (10.0 %)
left lobe	4/50 (8.0 %)
right lobe	20/50 (40.0 %)
bilobar; no data	17/50 (34.0 %); 9/50 (18.0 %)
infiltrative growth	11/50 (22.0 %)
nodular growth	36/50 (72.0 %)
infiltrative & nodular g.	3/50 (6.0 %)

On inclusion, 28 patients had less than 4 HCC lesions (56.0 %) and 17 patients had 4 or more lesions (34.0 %, no data = 5). These lesions were in the left lobe in 4 patients (8.0 %), in the right lobe in 20 patients (40.0 %), and in both lobes in 17 patients (34.0 %; no data = 9). Infiltrative tumor growth was observed in 11 patients (11.0 %), a nodular growth pattern in 36 patients (72.0 %) and an infiltrative as well as nodular growth pattern in 3 patients (6.0 %).

### DSM-TACE

DSM-TACE was used in a palliative treatment setting in 41 out of 50 cases (82.0 %). In five cases (10.0 %), chemoembolization was initially intended as a curative treatment. In two cases (4.0 %), it was applied before patients were listed for liver transplantation (no data = 1).

Application of DSM-TACE was conducted selectively via A. hepatica propria in 7 procedures (3.9 %), A. hepatica dextra in 48 procedures (26.8 %), both A. hepatica dextra and sinistra in 25 procedures (14.0 %), A. hepatica sinistra in 15 procedures (8.4 %), superselective catheterization in 45 procedures (25.1 %), both superselective catheter placement and selective catheterization of the A. hepatica dextra in 15 procedures (8.4 %) and A. hepatica dextra, A. hepatica sinistra and superselective catheterization in 21 procedures (11.7 %, no data = 3; Table 2).

**Table 2:** Details on the TACE procedures

Catheter position	
A. hepatica propria (AHP)	7/179 (3.9%)
A. hepatica dextra (AHD)	48/179 (26.8 %)
A. hepatica sinistra (AHS)	15/179 (8.4 %)
AHD + AHS	25/179 (14.0 %)
AHD + AHD + superselective	21/179 (11.7 %)
AHD + superselective	15/179 (8.4 %)
superselective; no data	45/179 (25.1 %); 3/179 (1.7%)
DSM dose (mg)	353.6 ± 140.1
Contrast agent (ml)	66.1 ± 60.1
Injection time of CTX (min)	22.9 ± 17.6
TACE sequence	
CTX and DSM combined	172/179 (96.1 %)
CTX prior to DSM	1/179 (0.6 %)
DSM prior to CTX; no data	3/179 (1.7 %); 3/179 (1.7 %)

A mean of 353.6 ± 140.1 mg of DSM was used per treatment, and 66.1 ± 60.1 ml of contrast agent was added. Application of chemotherapeutic agents spanned over 22.9 ± 17.6 minutes. DSM was given mixed with chemotherapeutic agents in 172 procedures, following chemotherapeutic agents in 1 case and prior to chemotherapeutic agents in 3 cases (no data = 3).

Doxorubicin, Epirubicin, and Carboplatin were used as the sole chemotherapeutic agent in 174 cases (Table 3, no data = 5). Doxorubicin was used in 84 cases with dosages from 25 to 50 mg (50 mg n = 70), Epirubicin in 87 cases with dosages from 20 to 100 mg, and Carboplatin in 3 cases with either 300 mg (n = 2) or 450 mg (n = 1).

**Table 3:** Details on the chemotherapeutic agents used

Chemotherapeutic agent	Dosage (mg)	n
Doxorubicin	25	10
	34, 5	1
	40	3
	50	70
Epirubicin	20	1
	25	1
	30	4
	50	52
	55	5
	60	21
	100	2
	no data	1
Carboplatin	300	2
	450	1

### Safety

In 2 patients, treatment had to be discontinued because of an allergic reaction to DSM during the first chemoembolization. Within 24 hours of DSM-TACE in 45 treatments (25.2 %), epigastric pain was registered as an immediate AE. Nausea and vomiting occurred after 20 treatments (11.2 %). After 2 treatments (1.1 %), dyspnea occurred, while after 1 treatment (0.6 %), shivering occurred. After 7 treatments (3.9 %), elevated temperature was reported. All adverse events were attributable to 26 of 50 patients. Thus, 24 patients (48.0 %) did not encounter any adverse events, 24 (48.0 %) had minor adverse events (AE), and 2 (4.0 %) had severe adverse events (SAE).

**Figure 1:**
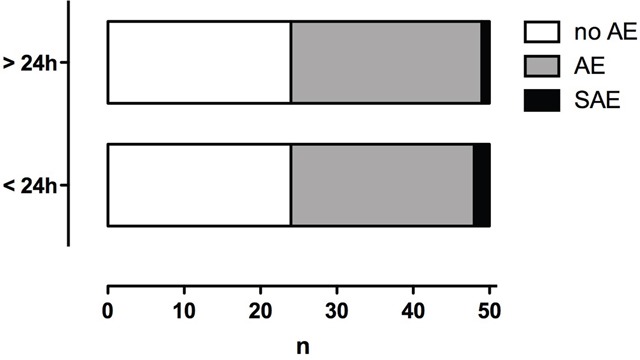
Number of patients affected by adverse (AE) and severe adverse events (SAE) Immediate events (< 24 h from DSM-TACE) and delayed events (> 24 from DSM-TACE).

Between DSM-TACE treatments, epigastric pain was registered in 27 cases and nausea and vomiting in 26 cases. After 7 procedures, elevated temperature was reported. In 2 cases, diarrhea occurred. One patient developed a gastric ulcer. Two treatments were followed by transient neutropenia and one by transient thrombopenia. These adverse events were attributable to 26 of 50 patients. In summary, AE occurred in 25 out of 50 patients (50.0 %), and one patient (2.0 %) had a SAE (Figure 1).

### Efficacy and tumor response

**Figure 2:**
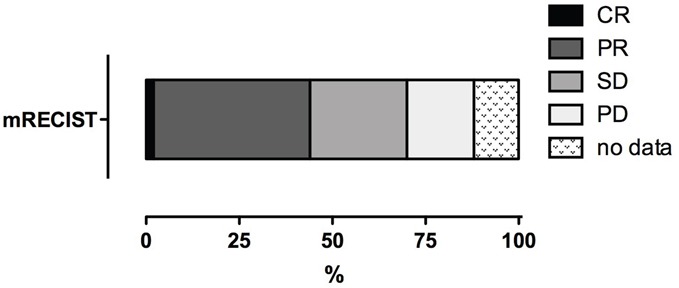
Tumor response of HCC to DSM-TACE CR = complete response, PR = partial response, SD = stable disease, PD = progressive disease.

Using the mRECIST criteria for the therapeutic efficacy assessment, the results were as follows: CR = 2 % (n=1), PR = 42 % (n=21), SD = 26 % (n=13), and PD = 18 % (n=9); for 12 % of patients (n=6), the follow up data were incomplete (Table 4). Thus, the objective response (OR) was 44 % (n=22) and disease control (DC) was 70 % (n=35; Figure 2).

**Table 4:** Final tumor response grading using mRECIST

	mRECIST
complete remission	1 (2 %)
partial remission	21 (42 %)
stable disease	13 (26 %)
progress	9 (18 %)
no data	6 (12 %)
objective response	22 (44 %)
disease control	35 (70 %)

## DISCUSSION

HCC is the 3^rd^ most common cause of cancer-related death worldwide [1]. Patients and medical professionals often face the challenge of the disease no longer being eligible for curative resection at the time of diagnosis. TACE has proven to be a safe and efficient therapeutic option in treating intermediate stage HCC [2]. It can be both a stand-alone treatment or bridge to other therapies, including resection or transplantation, if sufficient reduction of tumor burden can be realized. TACE comprises a vast field of setups with respect to its two major components: the chemotherapeutic agent and embolic agent. The latter is of major importance for the TACE procedure. The embolic agent delivers the chemotherapeutic agent to the target tissue and causes a vascular occlusion, which is a key component in TACE treatment. First, reduced blood flow extends the exposure time to CTX [7]. Second, the reduced wash out ramps up the locoregional concentration of CTX [7]. Third, ischemia can cause tumor necrosis [13]. Ischemia also causes changes to the cellular signaling pathways, possibly facilitating CTX uptake [14]. Finally, ischemia may facilitate the cytotoxic action of CTX by creating an oxygen-deprived environment that is favorable for free-radical formation [15]. DSM microspheres represent a major class of degradable embolic agents besides conventional Lipiodol and permanent occlusive drug eluting beads. Conventional TACE using Lipiodol has two major disadvantages. First, the lipophilic property reduce its capacity for loading with most chemotherapeutic agents, which are predominantly hydrophilic [16]. Additionally, it has been shown to be associated with a remarkable systemic VEGF (vascular endothelial growth factor) response, which may facilitate tumor growth and metastatic seeding [17, 18], which is possibly due to occlusion of the smallest caliber vessels, causing a characteristic ischemia-reperfusion (I/R) timeframe [14, 19]. Both DSM and DEB do not elicit this type of dominant VEGF response. Most microspheres and beads are 50 microns or larger, sparing the smallest vessels. With respect to the characterization of I/R, one must consider the unique anatomic properties of the liver with the dual blood supply, which is dominated by the portal vein (approx. 80%) and complemented by the hepatic artery (approx. 20%). Furthermore, this demands a clear and strict separation from the observation and evaluation of primary liver cancers, such as HCC with a hypervascular architecture, and secondary liver malignancies, such as colorectal metastasis. With respect to the combined results (metastases and HCC) published by Sun et al. for HepaSphere microspheres (Merit Medical Systems Inc., South Jordan, UT, USA) [20], our results showed a promising objective response rate of 44 %, and disease control rate of 70 %, which is worthy of further evaluation in randomized clinical trials. The safety and toxicity profile of Embocept DSM-TACE was very favorable in our cohort, which was composed of 179 DSM-TACE procedures in 50 patients with intermediate stage, non-resectable HCC. Kirchhoff et al. reported partial remission in 36 % of patients and no complete remission using degradable starch microspheres and Lipiodol [21]. In 2014, Malagari et al. reported an objective response rate of 68.9 % using DSM-TACE [16]. Largely, the results of TACE-treatment in liver malignancies vary widely. This is due, but not limited, to non-standardized TACE procedures and applications, different embolic agents, different chemotherapeutic agents, different pre-treatments, varying treatment concepts (stand-alone TACE vs. bridging), small cohort sizes, predominant single-center studies, different tumor entities and tumor burden. Further, multi-center randomized clinical trials must be performed to compare strict, standardized TACE-procedures to gain further insight into which embolic/chemotherapeutic agent combination is best for the treatment of HCC.

To specifically gain insights on EmboCept as an embolization agent in DSM-TACE, our study had the following limitations: it was set up as a solely observational study with non-standardized DSM-TACE procedures, including injection and combination of chemotherapeutics, non-strict inclusion and exclusion criteria in a multicenter design, as well as a loose imaging protocol. This study consequently lacked data and dedicated analyses on clinical outcome parameters, such as the local control rate, progression free survival, and overall survival. For a solely observational study, we accepted these limitations in favor of making promising results on the safety and efficacy of DSM-TACE as an available treatment option.

In conclusion, from the data presented here, EmboCept DSM-TACE seems to be a safe treatment option in intermediate stage HCC, and the results regarding its efficacy are promising. Nonetheless, further studies with strict protocols and long-term follow-ups comparing cTACE or DEB-TACE are required.

## MATERIALS AND METHODS

### Patients

Patients older than 18 years with confirmed HCC of intermediate stage that was not suitable for resection or any other curative treatment strategy were eligible for inclusion in this study. Both, nodular and infiltrative growth, as well as single-lobar and bilobar disease, were suitable for inclusion. Patients with prior chemoembolization, enrollment in any other clinical trial and/or other contraindications against DSM-TACE, such as lacking safe arterial access to the intrahepatic malignancy, were precluded from participation in the study.

Informed consent and approval by the local ethics committee were waived because the study was solely observational, and the study was conducted in accordance with the Declaration of Helsinki, the International Conference on Harmonization Guideline on Good Clinical Practice, and national laws and regulations where applicable.

### Study design

This was a national, multi-center observational study on the safety and efficacy of DSM-TACE for the treatment of intermediate HCC. The recruitment period for the study was from January 2010 to June 2014.

#### DSM-TACE

EmboCept S DSM 35/50 is a short-term embolizate that is composed of degradable starch microspheres with an average diameter of 50 micrometers. The microspheres are enzymatically degraded by serum alpha-amylases in the blood, yielding a half-life of approximately 35-50 minutes, both *in vivo* and *in vitro*. The resulting glucose fragments are further degraded by the reticulocyte system. A specific side-effect of the transient vascular occlusion strategies, such as in TACE, is the back-flow of the embolizate to non-target regions, causing ischemia and severe pain. DSM ameliorates the risk of profound organ damages due to its self-limiting degradation. With degradable starch microspheres, partial resumption of blood flow is observed after approximately 10 to 15 minutes [10].

After inclusion and imaging (MRI and/or CT), patients received DSM-TACE. Six weeks later, patients were restaged and received another cycle of DSM-TACE when suitable.

A catheter for DSM-TACE was placed via the femoral artery in all patients. Selective (A. hepatica propria, A. hepatica sinistra, A. hepatica dextra) or super-selective catheterization was subject to discretion by the interventionalist in charge depending on the tumor burden, vascular status, and individual patient and case characteristics.

The preparation and dosage of DSM was in line with the manufacturer's instructions. The choice of chemotherapeutic agent or a combination of up to three drugs was used depending on the individual patient and case characteristics. DSM allows for the use of different types of co-administered contrast agents. As in all TACE procedures, the order of application of the embolizate and chemotherapeutic agent was as follows: the embolizate was applied first, followed by the chemotherapeutic(s) or both in parallel.

#### Imaging

Within this observational study, the imaging modality for the staging of the intrahepatic lesions was chosen according to institution and/or interventionalist preference. Before and after DSM-TACE, all patients underwent CT or MR imaging using a multiphase liver imaging protocol. All patients underwent the same imaging modalities throughout their participation in the study to enable a reliable rating according to mRECIST.

#### Safety

A key point of the observational study reported here was to evaluate the DSM-TACE treatment-emergent side effects. The side effects were separately registered within 24 hours of the TACE-procedure (immediate). The side effects between single treatment sessions (delayed) were based on the CTCAE (Common Terminology Criteria for Adverse Events) V4.0 classification of acute and subacute toxic side effects, covering both serious adverse events (SAE) and adverse events (AE).

#### Efficacy

The efficacy of DSM-TACE in HCC treatment was rated according to mRECIST for the assessment of tumor necrosis in locoregional therapies [11, 12]. Complete response (CR) was defined as the disappearance of any intratumoral arterial enhancement in all target lesions. Partial response (PR) was defined as at least a 30% decrease in the sum of the diameters of viable (contrast enhancing) target lesions. Progressive disease (PD) was defined as an increase of at least 20% in the sum of the diameters of the viable (enhancing) target lesions, and stable disease included all cases that did not qualify as a either partial response or progressive disease. As previously reported [5], disease control (DC) was defined and calculated as CR + PR + SD. Objective response (OR) was defined and calculated as CR + PR.

### Statistical analysis

For statistical calculations, analysis, and plotting, we used GraphPad Prism version 5.00a for Mac (GraphPad Software, San Diego California, U.S.A.). Statistically significant differences were assumed at p <0.05.

## Abbreviations

AE: adverse event
AHD: A. hepatica dextra
AHP: A. hepatica propria
AHS: A. hepatica sinistra
CR: complete response
cTACE: conventional transarterial chemoembolization (with Lipiodol)
CTCAE: common terminology criteria for adverse events
CTX: chemotherapy
DC: disease control
DEB: drug eluting beads
DSM: degradable starch microspheres
HCC: hepatocellular carcinoma
mRECIST: modified response evaluation criteria in solid tumors
OR: objective response
PD: progressive disease
PR: partial response
RFA: radiofrequency ablation
SAE: severe adverse event
TACE: transarterial chemoembolization
VEGF: vascular endothelial growth factor

## References

[1] El-Serag HB (2011). Hepatocellular carcinoma. N Engl J Med.

[2] Llovet JM, Real MI, Montaña X, Planas R, Coll S, Aponte J, Ayuso C, Sala M, Muchart J, Solà R, Rodés J, Bruix J, Barcelona Liver Cancer Group (2002). Arterial embolisation or chemoembolisation versus symptomatic treatment in patients with unresectable hepatocellular carcinoma: a randomised controlled trial. Lancet.

[3] Llovet JM, Bruix J (2003). Systematic review of randomized trials for unresectable hepatocellular carcinoma: chemoembolization improves survival. Hepatology.

[4] Lencioni R, de Baere T, Soulen MC, Rilling WS, Geschwind JF (2016). Lipiodol transarterial chemoembolization for hepatocellular carcinoma: a systematic review of efficacy and safety data. Hepatology.

[5] Lammer J, Malagari K, Vogl T, Pilleul F, Denys A, Watkinson A, Pitton M, Sergent G, Pfammatter T, Terraz S, Benhamou Y, Avajon Y, Gruenberger T (2010). Prospective randomized study of doxorubicin-eluting-bead embolization in the treatment of hepatocellular carcinoma: results of the PRECISION V study. Cardiovasc Intervent Radiol.

[6] Vogl TJ, Lammer J, Lencioni R, Malagari K, Watkinson A, Pilleul F, Denys A, Lee C (2011). Liver, gastrointestinal, and cardiac toxicity in intermediate hepatocellular carcinoma treated with PRECISION TACE with drug-eluting beads: results from the PRECISION V randomized trial. AJR Am J Roentgenol.

[7] Varela M, Real MI, Burrel M, Forner A, Sala M, Brunet M, Ayuso C, Castells L, Montañá X, Llovet JM, Bruix J (2007). Chemoembolization of hepatocellular carcinoma with drug eluting beads: efficacy and doxorubicin pharmacokinetics. J Hepatol.

[8] Ebert M, Ebert J, Berger G (2013). Intravital microscopic research of microembolization with degradable starch microspheres. J Drug Deliv.

[9] Wiggermann P, Wohlgemuth WA, Heibl M, Vasilj A, Loss M, Schreyer AG, Stroszczynski C, Jung EM (2013). Dynamic evaluation and quantification of microvascularization during degradable starch microspheres transarterial Chemoembolisation (DSM-TACE) of HCC lesions using contrast enhanced ultrasound (CEUS): a feasibility study. Clin Hemorheol Microcirc.

[10] Håkansson L, Håkansson A, Morales O, Thorelius L, Warfving T (1997). Spherex (degradable starch microspheres) chemo-occlusion—enhancement of tumor drug concentration and therapeutic efficacy: an overview. Semin Oncol.

[11] Kim MN, Kim BK, Han KH, Kim SU (2015). Evolution from WHO to EASL and mRECIST for hepatocellular carcinoma: considerations for tumor response assessment. Expert Rev Gastroenterol Hepatol.

[12] Lencioni R, Llovet JM (2010). Modified RECIST (mRECIST) assessment for hepatocellular carcinoma. Semin Liver Dis.

[13] Huang X, Molema G, King S, Watkins L, Edgington TS, Thorpe PE (1997). Tumor infarction in mice by antibody-directed targeting of tissue factor to tumor vasculature. Science.

[14] Menger MD, Pelikan S, Steiner D, Messmer K (1992). Microvascular ischemia-reperfusion injury in striated muscle: significance of “reflow paradox.”. Am J Physiol.

[15] Meredith AM, Dass CR (2016). Increasing role of the cancer chemotherapeutic doxorubicin in cellular metabolism. J Pharm Pharmacol.

[16] Malagari K, Pomoni M, Moschouris H, Kelekis A, Charokopakis A, Bouma E, Spyridopoulos T, Chatziioannou A, Sotirchos V, Karampelas T, Tamvakopoulos C, Filippiadis D, Karagiannis E (2014). Chemoembolization of hepatocellular carcinoma with HepaSphere 30-60 μm. Safety and efficacy study. Cardiovasc Intervent Radiol.

[17] Poon RT, Lau C, Yu WC, Fan ST, Wong J (2004). High serum levels of vascular endothelial growth factor predict poor response to transarterial chemoembolization in hepatocellular carcinoma: a prospective study. Oncol Rep.

[18] Sergio A, Cristofori C, Cardin R, Pivetta G, Ragazzi R, Baldan A, Girardi L, Cillo U, Burra P, Giacomin A, Farinati F (2008). Transcatheter arterial chemoembolization (TACE) in hepatocellular carcinoma (HCC): the role of angiogenesis and invasiveness. Am J Gastroenterol.

[19] Liu K, Min XL, Peng J, Yang K, Yang L, Zhang XM (2016). The changes of HIF-1α and VEGF expression after TACE in patients with hepatocellular carcinoma. J Clin Med Res.

[20] Sun JH, Zhou GH, Zhang YL, Nie CH, Zhou TY, Ai J, Zhu TY, Wang WL, Zheng SS (2017). Chemoembolization of liver cancer with drug-loading microsphere 50-100μm. Oncotarget.

[21] Kirchhoff TD, Bleck JS, Dettmer A, Chavan A, Rosenthal H, Merkesdal S, Frericks B, Zender L, Malek NP, Greten TF, Kubicka S, Manns MP, Galanski M (2007). Transarterial chemoembolization using degradable starch microspheres and iodized oil in the treatment of advanced hepatocellular carcinoma: evaluation of tumor response, toxicity, and survival. Hepatobiliary Pancreat Dis Int.

